# High Serum Galectin-3 Level as a Potential Biomarker of Peripheral Artery Disease in Patients Undergoing Hemodialysis

**DOI:** 10.31083/j.rcm2504124

**Published:** 2024-03-29

**Authors:** Liang-Te Chiu, Bang-Gee Hsu, Yu-Hsien Lai, Chih-Hsien Wang, Jen-Pi Tsai

**Affiliations:** ^1^Division of Nephrology, Department of Internal Medicine, Dalin Tzu Chi Hospital, Buddhist Tzu Chi Medical Foundation, 62247 Chiayi, Taiwan; ^2^Division of Nephrology, Department of Internal Medicine, Hualien Tzu Chi Hospital, Buddhist Tzu Chi Medical Foundation, 97010 Hualien, Taiwan; ^3^School of Medicine, Tzu Chi University, 97004 Hualien, Taiwan

**Keywords:** C-reactive protein, hemodialysis, galectin-3, peripheral artery disease

## Abstract

**Background:**

Galectin-3 is implicated in the pathogenesis of inflammation and atherosclerosis. Peripheral arterial disease (PAD), characterized by a reduced ankle-brachial index (ABI), is a prognostic marker for mortality in patients on hemodialysis. We investigated the relationship between serum galectin-3 levels and PAD in patients undergoing regular hemodialysis.

**Methods:**

We carried out a cross-sectional study at a medical center, involving 92 participants. Serum galectin-3 levels were assessed by a commercially available enzyme-linked immunosorbent assay. ABI measurement was done with an automatic device based on oscillometry. Participants were categorized into two groups, normal and low ABI, based on a 0.9 cut-off point.

**Results:**

Eighteen patients (19.6%) exhibited a low ABI. In individuals with low ABIs, we observed a greater prevalence of diabetes mellitus, elevated serum C-reactive protein (CRP) levels, increased galectin-3 levels, and lower serum creatinine levels. Furthermore, serum galectin-3 levels (odds ratio [OR]: 1.056, 95% confidence interval [CI]: 1.003–1.112, *p* = 0.037) and CRP (per 0.1 mg/dL increment, OR: 1.195, 95% CI: 1.032–1.383, *p* = 0.017) were identified as independent predictors of PAD. Serum galectin-3 and log-transformed CRP levels were also independently and significantly negatively correlated with the left and right ABI values.

**Conclusions:**

Serum galectin-3 levels correlate with PAD in patients undergoing maintenance hemodialysis.

## 1. Introduction

Even with adequate hemodialysis, individuals with end-stage renal disease continue to experience a high rate of cardiovascular-related mortality [1, 2]. Peripheral artery disease (PAD) is an atherosclerotic condition affecting the lower limbs, typically defined by an ankle-brachial index (ABI) below 0.9 in either leg. PAD is linked to reduced physical activity, a heightened risk of limb amputation, and an increased likelihood of cardiovascular morbidity [3]. Individuals with PAD have a threefold higher risk of all-cause death and more than six times higher risk of dying from coronary heart disease when compared to those without this condition [3]. Patients undergoing hemodialysis (HD) face a significantly elevated risk of PAD, as evidenced by a meta-analysis that reported an average prevalence of 26.0%, in contrast to the general population, where it is no more than 10% [4]. Numerous studies have established a connection between PAD and death in HD patients [4, 5, 6]. One extensive meta-analysis showed that PAD substantially elevated the risk of death from any cause by over twofold and death from cardiovascular causes by nearly threefold in HD patients after adjusting for multiple variables [4]. Indeed, many asymptomatic HD patients with PAD may go undiagnosed, emphasizing the need for novel biomarkers to enable early detection and monitoring in this population.

Galectin-3 is a ubiquitously expressed β-galactoside-binding protein of the lectin family that regulates various cellular functions, such as cell-cell and cell-matrix interactions, proliferation, and differentiation [7]. Moreover, it contributes to the development of inflammation and tissue fibrosis [7, 8], and of conditions such as heart failure [9], atherosclerosis [10, 11], and cardiovascular diseases [12]. Human specimens showed increased galectin-3 levels in atherosclerotic carotid and lower limb arteries when compared to umbilical cord arteries [10]. Galectin-3 is considered a potential cardiovascular inflammatory biomarker [11]. According to the Atherosclerosis Risk in Communities (ARIC) study, an independent association was observed between galectin-3 and the development of incident PAD and critical limb ischemia in the general population [13]. Nonetheless, the relationship between galectin-3 concentration and PAD in patients receiving HD remains uncharted. To fill this void, our study was undertaken with the aim of investigating the connection between circulating galectin-3 concentration and PAD in patients on regular HD.

## 2. Methods

### 2.1 Patients

This cross-sectional investigation comprised 92 HD patients treated at an eastern Taiwan medical center from June 1, 2020, to August 31, 2020. Eligibility criteria included individuals aged 20 years or older who had undergone regular HD treatments lasting four hours, thrice weekly for at least six months (**Supplementary Fig. 1**). High-flux FX-class dialyzers (Fresenius Medical Care, Bad Homburg, Germany) were utilized in the dialysis procedures.

Demographic and medical information, which included data on diabetes mellitus (DM), hypertension, and smoking status, was extracted from the medical records of participants. Physician diagnosis or previous use of hypoglycemic or antihypertensive medication was employed to identify DM and hypertension, respectively. This study received approval from the Institutional Review Board of Tzu Chi Hospital and was conducted following the Helsinki Declaration guidelines (IRB108-219-A). Written informed consent was obtained from all participants. Patients were excluded through chart review and inquiry based on the following criteria: active cancer, acute infection within 30 days of enrollment, recent myocardial infarction, cerebrovascular event, or heart failure within 90 days of enrollment, amputation, refusal, or inability to cooperate. Additionally, patients taking cilostazol or pentoxifylline at the time of blood collection were excluded, along with those having elevated ABIs exceeding 1.3. High ABIs, typically exceeding 1.3 or 1.4, often indicate stiff lower limb arteries resistant to collapse by a pressure cuff, possibly due to vascular calcification [14].

### 2.2 Body Measurements

Body measurements were taken in the morning. Body mass index (BMI) was calculated by dividing body weight by the square of the height measurement [15, 16, 17].

### 2.3 Laboratory Analyses

Blood samples were gathered promptly prior to HD following an overnight fasting period. Serum levels of various parameters, including albumin, glucose, total cholesterol, blood urea nitrogen (BUN), creatinine, and C-reactive protein (CRP), were quantified (Siemens Advia 1800; Siemens Healthcare, Henkestr, Germany) [15, 16, 17]. Additionally, we measured serum galectin-3 (RayBiotech, Peachtree Corners, GA, USA) and intact parathyroid hormone (iPTH) (Abcam, Cambridge, MA, USA) using enzyme-linked immunosorbent assays [15, 16, 17]. The calculation of Kt/V index was based on the Daugirdas equation.

### 2.4 ABI Measurements

Blood pressure was assessed by oscillometry, with measurements taken three times on all four limbs. Patients lay flat on their back, and blood pressure was acquired from the arms and ankles by a Vascular Screening System VS-1000 device (Fukuda Denshi, Tokyo, Japan) [15, 16, 17]. The right ABI is determined by dividing the highest systolic ankle pressure measured from the right leg by the highest systolic brachial pressure in the arms, and the left ABI is calculated in the same way. Patients were diagnosed with PAD if their ABI was less than 0.9 on either side [15, 16, 17].

### 2.5 Statistical Analysis

The data were analyzed using the Statistical Package SPSS version 19.0 (SPSS Inc, Chicago, IL, USA). Continuous values were reported as mean ± standard deviation or median with 1st and 3rd quartiles, depending on their distribution. We compared means using an independent *t*-test and medians using the Wilcoxon rank-sum test. Categorical data were presented as counts with percentages and analyzed using the Chi-squared test. Subsequently, in a multivariable logistic regression analysis, we further evaluated the variables that exhibited significance (*p* < 0.2) in the aforementioned tests for their association with PAD. We conducted correlation analyses and multivariable stepwise linear regression analyses for both left and right ABI values. Additionally, we conducted receiver operating characteristic (ROC) analyses to calculate the area under the curve for serum galectin-3 and CRP concentrations to assess their diagnostic accuracy for PAD. To establish significance for all comparisons, the threshold was set at *p*-values less than 0.05.

## 3. Results

Baseline patient characteristics are displayed in Table 1. Among the 92 HD patients, 45 (48.9%) were female, the median HD duration was 51.24 months, and 15 (16.3%) were current smokers. Additionally, 37 (40.2%) had diabetes mellitus (DM), and 49 (53.3%) had hypertension. Mean serum galectin-3 level was 52.71 ± 14.90 ng/mL. Patients with low ABIs had a greater prevalence of DM (*p* = 0.011), elevated CRP and galectin-3 levels (both *p* < 0.001), and lower creatinine (*p* = 0.012), but no significant disparities in gender, hypertension prevalence, smoking, blood pressure, HD vintage, or Kt/V were observed between groups.

**Table 1.** **Clinical parameters in all patients and subgroups according to ABI**.

Characteristics		All Patients	Normal ABI	Low ABI	*p* value
		(*n* = 92)	(*n* = 74)	(*n* = 18)	
Age (years)		63.11 ± 13.46	62.43 ± 12.99	65.89 ± 15.32	0.331
Women, *n* (%)		45 (48.9)	36 (48.6)	9 (50.0)	0.918
Body mass index (${\mathrm{kg/m}}^{2}$)		24.82 ± 5.21	24.71 ± 5.01	25.30 ± 6.09	0.668
Comorbidities					
	Diabetes mellitus, *n* (%)	37 (40.2)	25 (33.8)	12 (66.7)	0.011*
	Hypertension, *n* (%)	49 (53.3)	41 (55.4)	8 (44.4)	0.403
	Current smoker, *n* (%)	15 (16.3)	10 (13.5)	5 (27.8)	0.142
Ankle brachial index					
	Left	1.06 ± 0.14	1.11 ± 0.09	0.84 ± 0.13	<0.001*
	Right	1.06 ± 0.14	1.10 ± 0.08	0.86 ± 0.13	<0.001*
Blood pressure					
	Systolic (mmHg)	144.79 ± 27.23	145.85 ± 27.89	140.44 ± 24.56	0.453
	Diastolic (mmHg)	77.79 ± 15.78	78.76 ± 16.58	73.83 ± 11.49	0.237
	HD vintage (months)	51.24 (21.33–122.16)	57.00 (21.51–130.21)	45.78 (20.94–77.67)	0.513
	Kt/V	1.36 ± 0.18	1.36 ± 0.17	1.36 ± 0.22	0.996
Laboratory data					
	Albumin (g/dL)	4.15 ± 0.47	4.19 ± 0.45	3.99 ± 0.55	0.124
	Glucose (mg/dL)	135.50 (110.00–169.00)	136.50 (111.75–168.25)	125.00 (90.25–286.75)	0.992
	Total cholesterol (mg/dL)	145.46 ± 37.12	147.81 ± 37.30	135.78 ± 35.72	0.219
	Blood urea nitrogen (mg/dL)	58.98 ± 13.87	60.26 ± 13.31	53.72 ± 15.25	0.073
	Creatinine (mg/dL)	9.22 ± 1.91	9.46 ± 1.94	8.21 ± 1.43	0.012*
	Intact parathyroid hormone (pg/mL)	255.24 ± 181.60	269.93 ± 184.10	194.86 ± 161.87	0.116
Biomarkers of inflammation					
	C-reactive protein (mg/dL)	0.29 (0.10–0.72)	0.24 (0.08–0.58)	0.79 (0.41–1.64)	<0.001*
	Galectin-3 (ng/mL)	52.71 ± 14.90	49.85 ± 13.47	64.45 ± 15.12	<0.001*

Continuous values were reported as mean ± standard deviation or median with 1st and 3rd quartiles. We compared means using an independent *t*-test and medians using the Wilcoxon rank-sum test; Categorical data were presented as counts with percentages and analyzed using the Chi-squared test. ABI, ankle brachial index; HD, hemodialysis; Kt/V, quality of dialysis. * *p* < 0.05 indicated statistical significance.

In the multivariable logistic regression analysis, both the serum CRP (odds ratio per 0.1 mg/dL increment: 1.195, 95% CI: 1.032–1.383, *p* = 0.017) and galectin-3 (odds ratio: 1.056, 95% CI: 1.003–1.112, *p* = 0.037) were identified as independent predictors of PAD. These associations were determined after accounting for potential confounding variables (*p* < 0.2), including DM, smoking status, albumin, blood urea nitrogen (BUN), creatinine, and iPTH (Table 2).

**Table 2.** **Multivariable logistic regression model for peripheral arterial disease and associated variables**.

Variables	Odds ratio	95% CI	*p* value
C-reactive protein, mg/dL	${\mathrm{1.195}}^{\text{a}}$	1.032–${\mathrm{1.383}}^{\text{a}}$	0.017*
Galectin-3, ng/mL	${\mathrm{1.056}}^{\text{b}}$	1.003–${\mathrm{1.112}}^{\text{b}}$	0.037*
Diabetes mellitus, yes	2.215	0.502–9.785	0.294
Current smoker, yes	3.597	0.418–28.849	0.228
Albumin, g/dL	${\mathrm{1.485}}^{\text{b}}$	0.165–${\mathrm{13.372}}^{\text{b}}$	0.724
Blood urea nitrogen, mg/dL	${\mathrm{0.979}}^{\text{b}}$	0.927–${\mathrm{1.034}}^{\text{b}}$	0.453
Creatinine, mg/dL	${\mathrm{0.696}}^{\text{b}}$	0.422–${\mathrm{1.147}}^{\text{b}}$	0.155
Intact parathyroid hormone, pg/mL	${\mathrm{1.000}}^{\text{b}}$	0.996–${\mathrm{1.004}}^{\text{b}}$	0.968

The analysis was conducted using multivariable logistic regression, with factors being diabetes, current smoker, albumin, blood urea nitrogen, creatinine, intact parathyroid hormone, C-reactive protein, and galectin-3. CI refers to confidence intervals. *Statistical significance was indicated by *p* < 0.05. ^a^ Represents odds ratio and 95% CI per 0.1 unit increment. ^b^ Represents odds ratio and 95% CI per 1 unit increment.

**Fig. 1.**
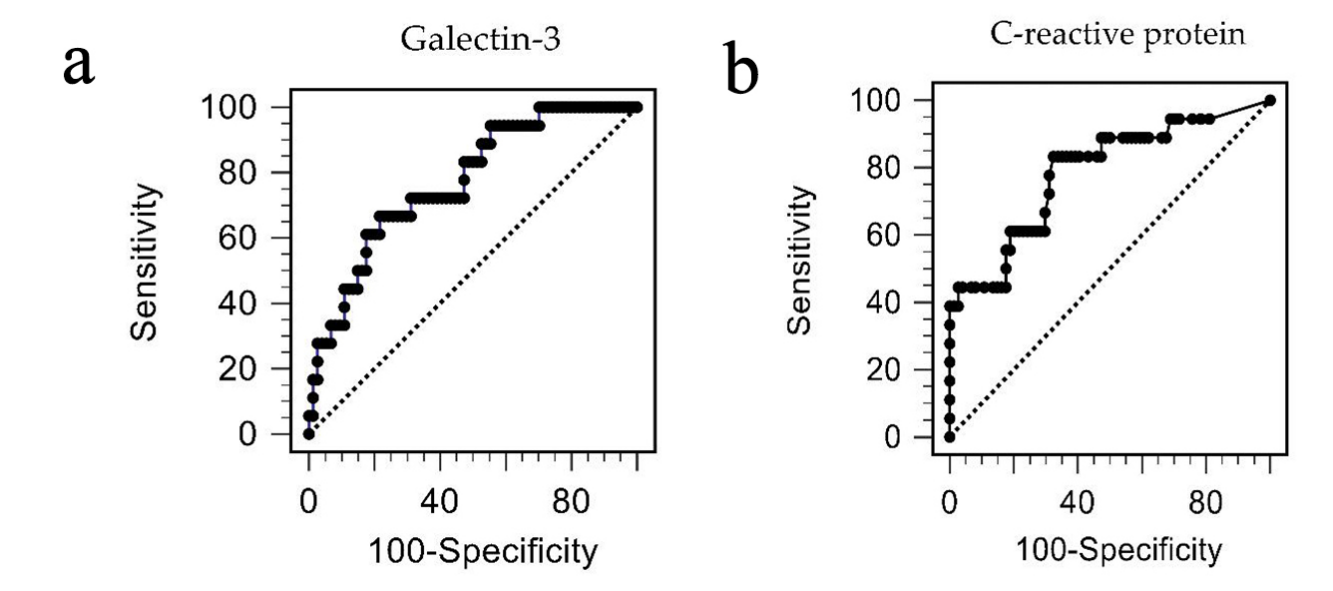
**The receiver operating characteristic curve demonstrates the accuracy of galectin-3 (a) and C-reactive protein (b) in diagnosing peripheral arterial disease**.

ROC analysis for diagnosis of PAD showed that the area under the curve was 0.771 (95% CI, 0.672–0.852; *p* < 0.001) for galectin-3 and 0.786 (95% CI, 0.688–0.864; *p* < 0.001) for CRP (Fig. 1a,b).

**Fig. 2.**
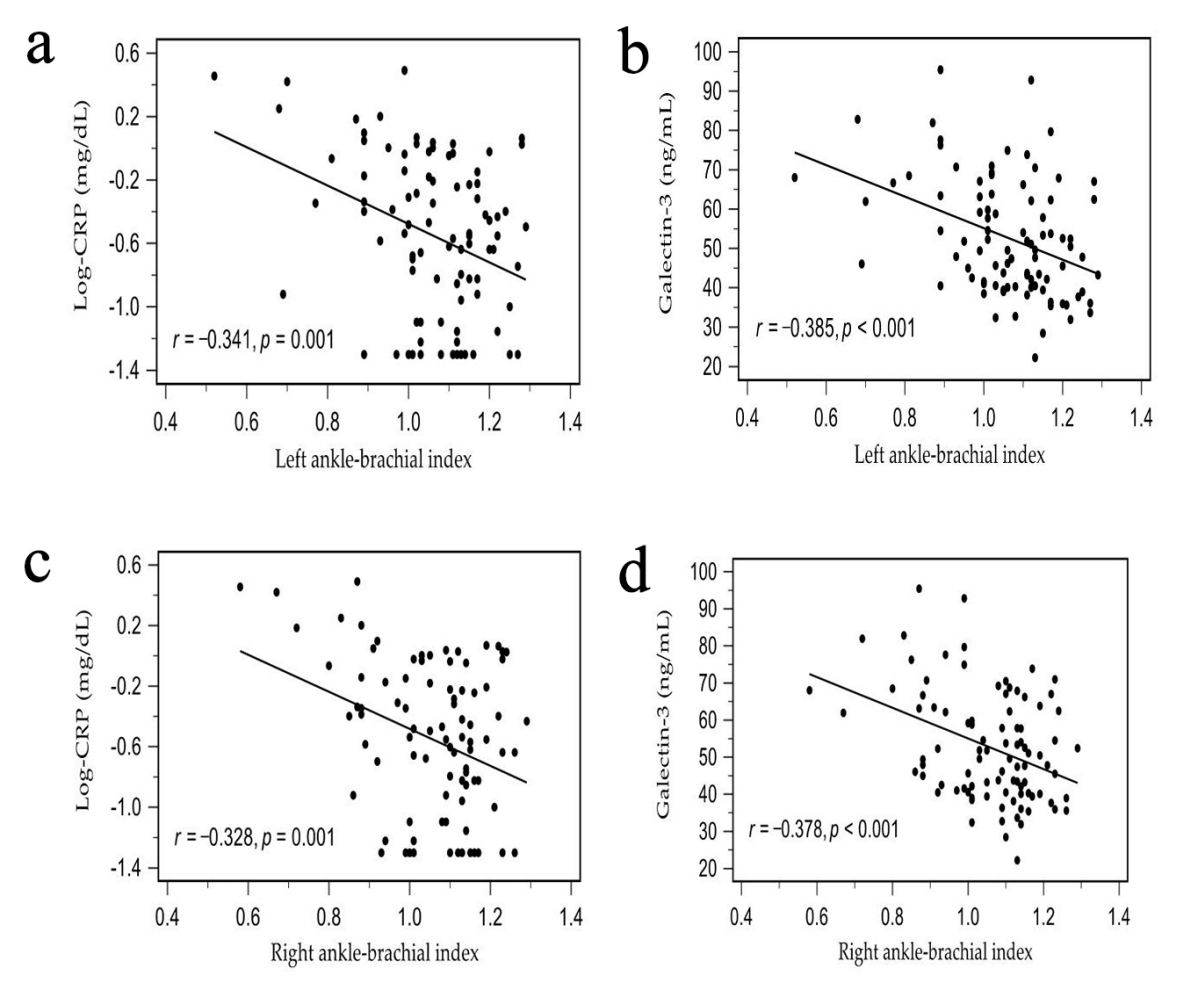
**Scatter plots of log-CRP (a) and galectin-3 (b) levels with left ABI values and log-CRP (c) and galectin-3 (d) levels with right ABI values among HD patients**. ABI, ankle brachial index; HD, hemodialysis; CRP, C-reactive protein.

Left and right ABI values were positively associated with serum creatinine and negatively associated with log-transformed CRP (log-CRP) and galectin-3 levels. Furthermore, left ABI values displayed a positive association with BUN, while right ABI values were negatively correlated with age (refer to Table 3). The multivariate stepwise linear regression model demonstrated significant correlations between log-transformed CRP (β = –0.245, adjusted coefficient of determination ($R^{2}$) change = 0.045, *p* = 0.016) and galectin-3 (β = –0.309, adjusted $R^{2}$ change = 0.139, *p* = 0.003) with left ABI values (refer to Table 4). Similarly, log-transformed CRP (β = –0.232, adjusted $R^{2}$ change = 0.040, *p* = 0.023) and galectin-3 (β = –0.305, adjusted $R^{2}$ change = 0.133, *p* = 0.003) were significantly associated with right ABI values (refer to Table 4). Two-dimensional scatterplots illustrating these correlations are presented in Fig. 2a–d.

**Table 3.** **Correlation analysis of left or right ankle-brachial index and clinical variables**.

Variables		Simple linear regression			
		Left ankle-brachial index		Right ankle-brachial index	
		r	*p* value	r	*p* value
Age, years		−0.122	0.245	−0.206	0.049*
Women		0.125	0.237	0.064	0.541
Body mass index, ${\mathrm{kg/m}}^{2}$		−0.143	0.175	−0.122	0.248
Diabetes mellitus		−0.194	0.064	−0.178	0.090
Current smoker		−0.111	0.292	−0.085	0.423
Blood pressure, mmHg					
	Systolic	0.116	0.272	0.038	0.719
	Diastolic	0.146	0.166	0.170	0.105
Log-HD vintage, months		0.045	0.671	0.002	0.984
Kt/V		0.153	0.145	0.170	0.104
Albumin, g/dL		0.112	0.289	0.062	0.557
Total cholesterol, mg/dL		0.137	0.194	0.155	0.140
Blood urea nitrogen, mg/dL		0.267	0.010*	0.168	0.109
Creatinine, mg/dL		0.238	0.022*	0.218	0.037*
Intact parathyroid hormone, pg/mL		0.110	0.298	0.137	0.192
Log- CRP, mg/dL		−0.341	0.001*	−0.328	0.001*
Galectin-3, ng/mL		−0.385	<0.001*	−0.378	<0.001*

We log-transformed skewed data, including HD vintage, and CRP levels, before conducting the analysis. Pearson or Spearman correlation analysis was chosen as deemed appropriate. HD, hemodialysis; CRP, C-reactive protein; Kt/V, quality of dialysis. * *p* < 0.05 indicated statistical significance.

**Table 4.** **Multivariable linear regression model of left or right ankle-brachial index and clinical variables**.

Multivariable linear regression			
Variables	Left ankle-brachial index		
	Beta	Adjusted $R^{2}$ change	*p* value
Log-CRP, mg/dL	−0.245	0.045	0.016*
Galectin-3, ng/mL	−0.309	0.139	0.003*
Variables	Right ankle-brachial index		
	Beta	Adjusted $R^{2}$ change	*p* value
Log-CRP, mg/dL	−0.232	0.040	0.023*
Galectin-3, ng/mL	−0.305	0.133	0.003*

Data analysis involved the use of multivariable stepwise linear regression, with included variables being age, creatinine, log-CRP, and galectin-3. CRP, C-reactive protein. * *p* < 0.05 indicated statistical significance.

## 4. Discussion

In this analysis involving 92 HD patients, we found a notable independent association between elevated serum galectin-3 levels and a heightened risk of PAD, even after accounting for variables such as DM and CRP. These results highlight the potential of galectin-3 as a marker for PAD in patients undergoing HD.

PAD ranks as the third most common cause of atherosclerotic cardiovascular morbidity, behind coronary artery disease and stroke [18]. Among patients suffering from PAD, the occurrence of coronary artery disease varies from 20% to 90%, depending on the diagnostic methods employed. Similarly, cerebrovascular disease has been identified in approximately 40% to 50% of PAD patients [3]. PAD is a surrogate marker for atherosclerotic disease, typically associated with risk factors like old age, DM, hyperlipidemia, smoking, and hypertension [3, 18]. In our study of chronic HD patients, among the previously mentioned traditional risk factors, only DM was associated with PAD, highlighting that conventional cardiovascular risk factors have limited predictive value in this population. This underscores the unique pathogenesis of atherosclerosis in individuals with uremia, consistent with existing knowledge that these classical risk factors incompletely explain the increased cardiovascular morbidity in advanced kidney disease [19, 20].

Atherosclerosis is more pronounced in individuals with renal failure, characterized by increased atherosclerotic plaque formation and calcification in the intimal layer, as well as extensive calcifications in the medial layer of arteries. These changes lead to vessel lumen narrowing, reduced elasticity, increased arterial stiffness, and ultimately contribute to significant cardiovascular-related diseases and fatalities in patients with advanced chronic kidney disease [21]. Proposed mechanisms include the accumulation of uremic toxins, inflammation, malnutrition, and increased oxidative stress [19, 22]. Inflammation is now widely acknowledged as a fundamental mechanism in the progression of atherosclerosis, and chronic kidney disease is characterized by a state of systemic inflammation. This condition is marked by elevated levels of circulating inflammatory cytokines, which activate macrophages and vascular endothelial cells, ultimately contributing to atherogenesis [19]. In our study, we observed that the PAD group had notably higher serum CRP levels and lower serum creatinine levels. These findings align with the understanding that inflammation and malnutrition are significant risk factors for atherosclerosis among individuals on dialysis. Lower serum creatinine levels likely indicate reduced muscle mass and, consequently, poorer overall nutritional status and even the presence of protein-energy wasting syndrome, as suggested by previous studies in patients on chronic hemodialysis [23].

Galectin-3 is now recognized as both a cardiovascular inflammatory biomarker and a mediator of atherosclerosis [11]. It induces the expression of various pro-inflammatory mediators in human macrophages [24]. Furthermore, Galectin-3 contributes to atherosclerosis by engaging in processes like lipid uptake, vascular smooth muscle cell proliferation and migration, and endothelial dysfunction [11, 12, 25, 26]. Oyenuga *et al*. [27] found that elevated galectin-3 levels correlate with increased carotid atherosclerosis detected through ultrasonography. A 10-year cohort study comprising 7968 Caucasians indicated that higher galectin-3 levels were connected to cardiovascular disease [28]. In the ARIC study, which included 9851 Americans over a median follow-up of 17.4 years, both galectin-3 and CRP were found to be associated with PAD incidence, emphasizing the role of fibrosis and inflammation in PAD development [13]. Associations between galectin-3 [29], CRP [30], and PAD were also reported in patients with diabetes. Our study further supports this concept in the HD population.

Serum galectin-3 levels rise as renal function declines, increasing by four to fivefold in dialysis patients [31]. Zhang *et al*. [32] utilized pulse-wave velocity measurements to demonstrate the correlation between galectin-3 and arterial stiffness in HD patients. Ursli *et al*. [33] found an inverse correlation between serum galectin-3 levels and the ABI in individuals with chronic kidney disease. Furthermore, research has consistently indicated that elevated galectin-3 levels are linked to adverse cardiovascular outcomes in both chronic kidney disease patients and those on dialysis [31, 34]. Zhang *et al*. [35] conducted a study indicating that combining pulse-wave velocity and galectin-3 can predict cardiovascular complications in HD patients. Our study aligns with prior research by demonstrating that galectin-3 and CRP are associated with ABI values and serve as independent risk markers for PAD among HD patients, as shown through multivariable logistic regression analysis. Additional research is needed to confirm whether serum galectin-3 concentration could predict cardiovascular morbidity in individuals with renal failure.

This study had certain limitations. Firstly, this study is limited by relying solely on ABI measurements for PAD diagnosis, lacking data on clinical symptoms or physical examinations. ABI may underestimate PAD prevalence in HD patients due to vascular calcification. Future studies should consider additional diagnostic measures, such as toe brachial index measurements alongside ABI [14, 36]. Secondly, it was a cross-sectional study conducted at a single medical center with a relatively modest sample size. Therefore, larger scale longitudinal studies are needed to validate the relationship between galectin-3 and PAD in HD patients, potentially establishing causality.

## 5. Conclusions

Serum galectin-3 and CRP are independently and positively linked to PAD in maintenance HD patients, regardless of traditional cardiovascular predictors. These results highlight the significance of galectin-3 and CRP, which are indicative of fibrosis and inflammation, in the development of PAD among HD patients.

## Data Availability

The data presented in this study can be requested from the corresponding author.

## Supplementary Material

Supplementary material associated with this article can be found, in the online version, at https://doi.org/10.31083/j.rcm2504124.
