# Supplementary material for: High Serum Galectin-3 Level as a Potential Biomarker of Peripheral Artery Disease in Patients Undergoing Hemodialysis
Source: Rev Cardiovasc Med. 2024 Mar 29;25(4):124. doi: 10.31083/j.rcm2504124 (PMC11263986; doi:10.31083/j.rcm2504124)
Supplement: Supplementary file 1 [file 2153-8174-25-4-124-s1.docx]

Patients included in the analysis
n= 92

 Excluded: n= 66

Active cancer, n= 6
acute infection, n= 4

recent MI/CVA/HF, n= 10
amputation, n= 6

refusal or inability to cooperate, n= 28
taking cilostazol/pentoxifylline, n= 7
and elevated ABIs> 1.3, n= 5

Normal ABI
( 0.9 ≤ABI ≤ 1.3)
n = 74

Patients aged 20 years or older, who were under regular HD treatment for at least six months between June and August of 2020
n= 158

(n= )

Low ABI
( ABI< 0.9 on either side)
n = 18

**Supplementary Fig. 1. Flow chart of patient inclusion and exclusion criteria.** MI, myocardial infarction; CVA, cerebrovascular accident; HF, heart failure.
